# Use of a Modified Pediatric Early Warning Score in a Department of Pediatric and Adolescent Medicine

**DOI:** 10.1371/journal.pone.0072534

**Published:** 2013-08-26

**Authors:** Anne L. Solevåg, Elisabeth H. Eggen, Judith Schröder, Britt Nakstad

**Affiliations:** 1 The Department of Pediatric and Adolescent Medicine, Akershus University Hospital, Lørenskog, Norway; 2 University of Oslo, Oslo, Norway; The Ohio State University, United States of America

## Abstract

**Background:**

Several versions of the Pediatric Early Warning Score (PEWS) exist, but there is limited information available on the use of such systems in different contexts. In the present study, we aimed to examine the relationship between a modified version of The Brighton Paediatric Early Warning Score (PEWS) and patient characteristics in a Norwegian department of pediatric and adolescent medicine. In addition, we sought to establish guidelines for escalation in patient care based on the PEWS in our patient population.

**Methods:**

The medical records of patients referred for acute care from March to May 2011 were retrospectively reviewed. Children with a PEWS ≥3 were compared to children with a PEWS 0–2 with regard to age, diagnostic group and indicators of severe disease.

**Results:**

A total of 761 patients (0−18 years of age) were included in the analysis. A younger age and diagnostic groups such as lower airway and cardiovascular disease were associated with PEWS ≥3. Upper airway disease and minor injury were more frequent in patients with PEWS 0−2. Children with PEWS ≥3 received fluid resuscitation, intravenous antibiotics, and oxygen supplementation, and were transferred to a higher level of care more often than children with PEWS 0−2.

**Conclusions:**

A PEWS ≥3 was associated with severe illnesses and surrogate markers of cardio-respiratory compromise. Patients with PEWS ≥3 should be carefully monitored to prevent further deterioration.

## Introduction

Over the past several decades, a number of scoring systems have been used to identify adults that are at risk for a deterioration in their condition. Many of these early warning scoring systems are based on clinical parameters such as systolic blood pressure, heart rate, respiratory rate, temperature, and neurologic status, as assessed by the ‘Alert, Voice, Pain, Unresponsive’ (AVPU) scale [1]. Several modifications and validations of the early warning score systems for adults have been published [1], [2], and a modified early warning score (MEWS) developed by Morgan et al. [3] is commonly used at present. However, this system and similar severity-of-illness scoring systems have not been validated for use in children.

Although structured observation and examination is as crucial in children as in adults [4], [5], [6], [7], knowledge on the manner in which pediatric early warning scoring systems can be used in different settings is limited. The first published report on the use of a pediatric early warning score was a short report by Monaghan in 2005, which described a 3-item tool for detecting clinical deterioration in children [8]. This scoring system was later referred to as the Brighton Paediatric Early Warning Score (PEWS), and was modified by Akre et al. [9] in 2010. The PEWS has several similarities to the MEWS. Unlike the MEWS, the PEWS does not include blood pressure and temperature measurements.

The rationale for using early warning scoring systems is that signs of deterioration have been shown to be present and detectable in many patients several hours before undergoing a serious life-threatening event [10], [11]. In addition, clinical signs of critical conditions are similar regardless of the underlying cause. When combined with the Airway, Breathing, Circulation, Disability, Exposure (ABCDE) approach, which should be used whenever critical illness or injury is suspected, early warning scores can help us detect and prevent deterioration in a patient’s condition.

### The Department of Pediatric and Adolescent Medicine, Akershus University Hospital

Akershus University Hospital (AHUS) is located just outside the Norwegian capital, Oslo. Although AHUS is the largest acute hospital in Norway, it has a relatively low patient turnover due to a smaller population size compared to hospitals in the US and Canada, where validation studies of different versions of the PEWS have been performed. In addition, severe traumatic injuries, as well as cardiac- or neuro-surgery are centralized to tertiary centers in Oslo. The hospital does not have a so-called rapid response team (RRT) or medical emergency team (MET). RRTs and METs are ambulatory outreach teams that assess patients with deteriorating physiology to establish measures aimed at preventing further deterioration, cardiac arrest, and death [12].

The Department of Pediatric and Adolescent Medicine has 112,000 patients with ages ranging from 0−18 years in its catchment area. Children who are treated by general surgeons, orthopedic surgeons, ear nose and throat (ENT) specialists, and pediatricians are all cared for by the same nurses in the children’s emergency department (ED) and in the wards. Hence, nurses and nurses’ assistants are faced with the challenges of assessing children with a wide range of conditions.

Children are referred to the ED by general practitioners who determine which patients require the immediate care of a specialist. Hence, there is a selection of patients who are more severely ill than those seen in the EDs of hospitals where referral is not required. Thus, as many as 50% of the patients seen in our ED require hospital admission. The rest of the patients referred for acute care are treated as outpatients.

Because of its geographical closeness to the large tertiary centers at Oslo University Hospital (OUH), AHUS does not have a pediatric intensive care unit (PICU), and transfers children below the age of 3 years who require intensive care to OUH. Critically ill children between 3 and 18 years are admitted to the intensive care unit for adults at AHUS.

In the present study, patients in our department were assessed using a slightly modified and translated version of the Brighton PEWS [8]. We aimed to assess the correlation of PEWS results with other indicators of severe illness (e.g. certain diagnostic groups and administration of cardio-respiratory support such as fluid resuscitation and supplementary oxygen) in a retrospective chart review. In addition, we attempted to establish a definition of critical PEWS in our patient population to determine PEWS values warranting increased attention to the patient.

## Materials and Methods

Because the purpose of the study was quality improvement the institutional review board of our hospital (Akershus University Hospital) declared that medical ethical review of the study was not required and thus waived the requirements for obtaining parental or guardian permission for performing a retrospective chart review.

In January/February 2011, the modified Brighton PEWS [9] was translated to Norwegian and the order of the 3 items (behavior, cardiovascular, and respiratory) was changed to match the well-known ABCD(E)-algorithm. In addition, the AVPU scoring system for the assessment of disability/behavior was incorporated (Table 1).

**Table 1:** The Brighton Paediatric Early Warning Score (PEWS) modified for use in our department.

	0	1	2	3
**Respiration**	Normal respiratory rate and SpO_2_	Respiratory rate ≥10 above normal parameters	Respiratory rate ≥20 above normal parameters	Respiratory rate ≥30 above normal parameters OR ≤5 below normal parameters
	**AND**	**OR**	**OR**	**AND**
**Airways**	No retractions	Retractions	Jugular retractions	Retractions or grunting
**Breathing**		**OR**	**OR**	**OR**
		FiO_2_>0.30 (CPAP/BiPAP)	FiO_2_>0.40 (CPAP/BiPAP)	FiO_2_>0.50 (CPAP/BiPAP)
		**OR**	**OR**	**OR**
		≥2 L/min O_2_	≥5 L/min O_2_	≥8 L/min O_2_
**Circulation**	Normal skin color	Pale	Grey or cyanotic	Grey or cyanotic AND mottled
	**OR**	**OR**	**OR**	**OR**
	Capillary refill time of 1–2 s	Capillary refill time of 3 s	Tachycardia, 20–30 beats/min above normal rate	Tachycardia, >30 beats/min above normal rate
			**OR**	**OR**
			Capillary refill time of 4 s	Bradycardia
				**OR**
				Capillary refill time of ≥5 s
**Disability**	Alert	Voice	Pain	Unresponsive

A maximum of 3 points can be assigned for each of 3 main components (respiration, circulation, and behavior/‘disability’). In addition, 2 additional points can be awarded if either continuous inhalation medications or continuous positive airway pressure (CPAP) treatments are being administered, and 2 additional points for the presence of persistent postoperative vomiting. Hence, the score ranges from 0 to 13, with zero representing a normal physiologic state.

The scoring system is divided into 1) respiratory, 2) circulatory, and 3) behavioral signs of clinical deterioration, which are scored on a scale from 0 to 3 for each parameter. The main components are respiratory rate, retractions, need for oxygen supplementation, heart rate, capillary refill time, skin color, and alertness. Respiratory rate and heart rate are assessed in relation to the normal range of values for different age categories, as defined by Akre et al. [9] (Table 2). Two additional ‘points’ can be awarded if either continuous inhalation medications or continuous positive airway pressure (CPAP) are being administered, and 2 additional points for the presence of persisting postoperative vomiting. Hence, the score ranges from 0 to 13 with zero representing a normal physiologic state.

**Table 2:** Age-specific physiological heart rate and respiratory rate at rest.

Age	Heart rate	Respiratory rate
	(beats per minute) at rest	(breaths per minute) at rest
0–1 months	100–180	40–60
1–12 months	100–180	35–40
13 months to 3 years	70–110	25–30
4–6 years	70–110	21–23
7–12 years	70–110	19–21
13–19 years	55–90	16–18

Nurses and nurses’ assistants in the department were instructed in the use of the PEWS during a one-day course in March 2011. All children referred for acute care from 15^th^ of March to 31^st^ of May 2011 were scored upon arrival in the children’s ED, and if admitted, 3 times every 24 hours (once every nurse shift). Respiratory rate, heart rate, capillary refill time etc. and the resulting PEWS scores of each child were recorded on paper forms. Because the aim of the study was not to validate the PEWS, but to measure utility, the study period was determined by convenience rather than statistical power calculation.

The PEWS forms obtained during the study months were collected from the ED and the wards. EHE who is a resident pediatrician checked the forms for erroneous scores. Scores were assessed and corrected in accordance with clinical information recorded in the electronic patient charts. If scores were incomplete or erroneous and could not be corrected retrospectively due to missing data, the whole PEWS form was excluded from analysis.

Patients were categorized into diagnostic groups based on the definition of Slater et al. for the categorization of transfers to intensive care units [13]: Injury (in our hospital: fractures and minor trauma); congenital cardiovascular disease; acquired cardiovascular disease; neurological disease including seizures and meningitis; renal disease including urinary tract infection; gastrointestinal disease; respiratory, upper airway diseases; respiratory, lower airway diseases including asthma, bronchiolitis, pneumonia, or pneumonitis; ‘other infection’; and miscellaneous including dehydration and diabetes ketoacidosis.

The highest PEWS for each patient was used for statistical calculations. Patients with the highest PEWS ≥3 were compared with patients with the highest PEWS ranging from 0 to 2 with respect to age, gender, diagnostic group, length of hospital stay, and different interventions as follows: Transfer to a higher level of care; oxygen supplementation; fluid resuscitation defined as a bolus of 10−20 mL crystalloid (sodium chloride or ringer acetate); intravenous (i.v.) antibiotics; i.v. rehydration defined as continuous crystalloid (sodium chloride or ringer acetate) or glucose infusion; rehydration via orogastric or nasogastric feeding tube; treated as inpatient or outpatient; and readmission within 30 days.

One investigator (ALS) categorized all the 761 forms in terms of diagnostic group and interventions. For a rough determination of inter-observer agreement, a second investigator (EHE) did the same categorization for the first 200 patients. The conclusions drawn by the two investigators were essentially the same. However, agreement was not measured by statistical calculations.

Statistical analyses were performed using IBM SPSS Statistics 20.0 for Mac (Armonk, New York, US). Descriptive statistics are presented as median with interquartile range (IQR) and comparisons between patients with PEWS ≥3 and patients with PEWS 0−2 were performed for continuous variables using the Mann-Whitney *U* test. Categorical data were compared using Fisher’s exact test. p-values <0.05 were considered significant.

## Results

### General

A total of 798 PEWS forms were collected. Of these, 37 were excluded because of insufficient data that could not be supplemented with documentation from the electronic patient chart.

Of the remaining 761 forms, 123 patients (16.2%) had a highest PEWS of ≥3 and 638 patients (83.8%) had a highest PEWS between 0 and 2. The characteristics of the patients with PEWS ≥3 and 0−2 are shown in Table 3. The highest PEWS identified was 7.

**Table 3:** Characteristics of the patients with a highest Pediatric Early Warning Score (PEWS) ≥3 compared to the patients with a highest PEWS of 0−2.

Patient characteristics	PEWS ≥3	PEWS 0–2	p-value
Age (years)*	2 (1.0–6.5)	4 (1.5–12)	0.001
Median (IQR)			
Sex (boy/girl)	71/52	338/300	0.19
Length of hospital stay (days)*	3 (2–5)	2 (1–3)	<0.001
Median (IQR)			
Oxygen supplementation*	18/123 (14.6%)	7/638 (1.1%)	<0.001
Fluid resuscitation*	9/123 (7.3%)	8/638 (1.3%)	<0.001
i.v. antibiotics*	24/123 (19.5%)	54/638 (8.5%)	<0.001
i.v. rehydration	20/123 (16.3%)	75/638 (11.8%)	0.11
Rehydration via feeding tube	4/123 (3.3%)	20/638 (3.1%)	0.56
Readmission	19/123 (15.4%)	94/638 (14.7%)	0.34
Inpatient*	107/123 (87.0%)	388/638 (60.8%)	<0.001

p<0.05 is considered a significant difference (marked with *).

### Age and Gender

We reviewed the charts of 409 boys and 352 girls. There was no difference in sex distribution between the PEWS groups (p = 0.33).

The median (IQR) age of the patients was 3.5 (1.2−11.0) years, with a range of 0−18 years for the 761 included patients. A proportion of 31.5% of the patients were aged between13 months and 3 years.

Patients with a PEWS ≥3 had a significantly lower median age than patients with PEWS 0−2 (Table 3).

### Interventions

Six (4.9%) of the patients with a PEWS ≥3 and 9 children (1.4%) with a PEWS 0−2 were transferred to a higher level of care, indicating that transfer to higher level of care was significantly more frequent among patients with PEWS ≥3 (p = 0.04).

Children with PEWS ≥3 received fluid resuscitation, oxygen supplementation, and i.v. antibiotics significantly more often than those with PEWS 0−2. However, no differences were detected in the requirement for i.v. rehydration or rehydration via an oro−/nasogastric feeding tube between the two groups (Table 3).

Patients with PEWS ≥3 had a higher proportion of admissions compared to patients with PEWS 0−2. There was no difference in the number of readmissions between the groups (Table 3).

### Diagnostic Groups and Discipline

The most prevalent diagnostic groups were gastrointestinal disease, respiratory (lower airway) disease, and neurological disease.

The distribution of the different diagnostic groups in patients with the highest PEWS ≥3 versus those with PEWS 0−2 is shown in Table 4. Lower airway disease was the diagnostic group with the highest fraction of patients with a PEWS ≥3. PEWS 0−2 was more often associated with gastrointestinal, renal, and upper airway diseases, as well as ‘other infections’ and injury, the latter of which was reflected by the increased proportion of PEWS 0−2 noted among patients treated by general and orthopedic surgeons. Only 1.1% of the surgical patients had a PEWS ≥3 compared to 20.6% of the medical patients (i.e. treated by pediatricians) (p<0.001).

**Table 4:** Distribution of diagnostic groups among the patients with a highest Pediatric Early Warning Score (PEWS) ≥3 compared to the patients with a highest PEWS of 0−2.

	PEWS ≥3	PEWS 0–2	p-value	Total
Injury*	3/123 (2.4%)	89/638 (13.9%)	<0.001	92/761 (12.1%)
Congenital cardiovascular disease*	5/123 (4.1%)	5/638 (0.8%)	<0.001	10/761 (1.3%)
Acquired cardiovascular disease*	4/123 (3.3%)	11/683 (1.6%)	<0.001	15/761 (2.0%)
Neurological disease*	8/123 (17.9%)	100/638 (11.8%)	0.007	108/761 (14.2%)
Renal disease including urinary tract infection*	3/123 (2.4%)	23/638 (3.6%)	<0.001	26/761 (3.4%)
Gastrointestinal disease*	15/123 (12.2%)	142/638 (22.3%)	0.011	157/761 (20.6%)
Respiratory, upper airway disease*	9/123 (7.3%)	71/638 (11.1%)	<0.001	80/761 (10.5%)
Respiratory, lower airway disease*	57/123 (46.4%)	54/638 (8.5%)	<0.001	111/761 (14.6%)
‘Other infection’*	8/123 (6.5%)	53/638 (8.3%)	<0.001	61/761 (8.0%)
Miscellaneous including dehydration and diabetes ketoacidosis	11/123 (8.9%)	90/638 (14.1%)	0.15	82/761 (10.8%)

p<0.05 is considered a significant difference (marked with *).

## Discussion

### Age

The patients with a PEWS ≥3 had a lower median age than the patients with PEWS 0−2. This finding is in agreement with data showing that the risk of cardio-respiratory arrest is higher in younger patients [14]. However, the association between a high PEWS and younger age could have many underlying causes, such as the emphasis on assessing signs of respiratory distress in this system: As the chest is more compliant in infants, this group is more likely to have retractions in the setting of respiratory diseases [15], and a PEWS ≥3 is therefore easier to attain. A certain degree of selection bias may also be present as one can expect that a larger number of severely ill children in the lower age categories are being referred to hospitals.

### Diagnostic Groups

In a prospective investigation of in-hospital pediatric cardiopulmonary resuscitation, Reis et al. concluded that 61% of pediatric cardiac arrests were caused by respiratory failure and 29% were caused by shock [16]. Cyanosis, poor peripheral circulation, rapid breathing, and shortness of breath were found to be predictors of severe illness in a low-prevalence study by Van den Bruel et al. [17]. In our study, patients with lower airway disease and cardiovascular disease (congenital and acquired) more frequently had a PEWS ≥3. As these diagnoses may predispose a child to respiratory and circulatory failure, respectively, our results suggest that the PEWS is elevated in high-risk patients.

In a systematic review by Thompson et al. [18], symptoms that are common in children, such as cough, abdominal pain, vomiting, diarrhea, poor feeding, and coryza, were found to be less alarming. We found that diagnostic groups including patients with these symptoms, such as upper airway and gastroenterological disease (including acute abdominal pain), were associated with a PEWS 0−2.

### Challenges

The PEWS is assessed using different charts, and hospitals may calculate PEWS in different ways, resulting in unreliable scoring. Moreover, there is limited research into how different scoring levels should lead to a range of actions.

Our experience with the AVPU scale for classifying consciousness suggests that we tend to underestimate the degree of somnolence and mental compromise in children at a very young age. For example, drowsy, uneasy children are often scored as being ‘Alert’ (0 points for behavior) because they are awake. Hence, the AVPU way of rating behavior (‘disability’) may be less sensitive than the classification of behavior in the original version of the Brighton PEWS [8] as such children might have received 3 points for ‘lethargic’ in the original version. Such adjustments to the PEWS may lead to other cut-off levels for escalation.

The effect of fever and crying on physiological parameters also poses a challenge for the interpretation of the PEWS. Therefore, re-evaluation of the PEWS in a feverish child is recommended after the administration of antipyretics.

### Context

There is limited information available on the use of the PEWS in different patient populations. This study was performed in a department of pediatric and adolescent medicine that mainly reflects the population, referral practices and organization of specialist healthcare in Norway and the Nordic countries. Although our unit is large for Norwegian standards, the number of patients seen in the ED is low in an international setting. In addition, because all patients seen in the ED have been referred by general practitioners, there is a selection of severely ill children compared to clinics where patients are seen without referral. Furthermore, the guidelines for providing intensive care and the thresholds for admission to intensive care units vary according to the context. Because of the need to transfer young patients requiring intensive care to a different hospital, the threshold is relatively high in our department. This may contribute to the low incidence of transfer to a higher level of care in our patient population. Due to the low number of ICU admissions it would probably take years to measure a statistically significant reduction in number of ICU admissions as a result of PEWS scoring (i.e. validating the PEWS in terms of avoiding ICU admissions) in our department.

As one of the main objectives of this paper was to describe how the modified PEWS relates to patient characteristics in a certain patient population, we devote attention to describing the context of the study elaborately. The purpose is to enable the reader to judge to what extent the results are relevant to children and adolescents in the context of the reader.

### Clinical Application

There are two possible uses for an early warning system such as the PEWS: (1) as a triage tool to identify the sickest children on presentation to the ED, and (2) as an early warning tool to identify the patient at risk of clinical deterioration (where a change from ‘baseline’ becomes particularly important).

Monaghan performed a pilot study of the Brighton PEWS in a 24-bed medical unit in which the PEWS was integrated into the daily routine of the medical staff [8]. In that study, a nurse calculated the PEWS of each child and depending on the score, one of four actions was taken:

1. The nurse in charge was informed
2. The frequency of observations was increased
3. A medical review was requested, and
4. The outreach team was informed or the full medical team and outreach team were called.

Akre et al. [9] performed a retrospective study using the modified Brighton PEWS in which children were identified before a critical event (rapid response callout or cardio-respiratory arrest) with a sensitivity of 85% using a cut-off critical PEWS of ≥4 or a single domain score of 3.

Skaletzky et al. [19] applied a further modified version of the Brighton PEWS and showed that the mean (SD) maximum (highest) PEWS in patients admitted to the PICU was 2.95 (1.5), as compared to 1.4 (0.8) in age- and diagnosis-matched controls. The sensitivity and specificity of a PEWS of 2.5 for transfer to a higher level of care were 62% and 89%, respectively.

In the present study, almost 5% of the patients with a highest PEWS ≥3 were transferred to a higher level of care. Together with the results of Akre et al. [9] and Skaletzky et al. [19], our findings suggest that a score ≥3 should indicate that the patient requires careful monitoring.

The lack of an RRT or a MET in our hospital prevented the assessment of the predictive value of a certain PEWS in relation to RRT or MET callouts. Furthermore, the proportion of patients transferred to the intensive care unit was low (2% of all included patients). In this respect, our results are not directly comparable to the published validation studies of the PEWS.

However, our results together with those of Monaghan, Akre, and Skaletzky [8], [9], [19] have led us to propose the following guidelines for our modified Brighton PEWS:

- All children should have their PEWS recorded in the emergency department, on admission to the ward and during the first hour of every nursing shift.
- The physician in charge should decide whether the PEWS should be assessed more frequently.
- The physician in charge should be informed about any increase in a child’s PEWS ≥2 or whenever the PEWS is 3 despite initiation of simple measures like antipyretics or bronchodilators.
- If the PEWS is 4, the physician in charge should attend to the child within 30 minutes.
- A PEWS ≥5 should lead to evaluation by both the physician in charge and an anesthesiologist.

Documentation of vital signs is often incomplete. In a retrospective study by Ludikhuize et al., respiratory rate, diuresis, and oxygen saturation were documented in only 30−66% of assessments, even when the MEWS was ≥3 [20].

The PEWS constitutes a platform for the objective evaluation of the condition of a child by allowing the conversion of routine observations into an actionable index that provides the basis for further evaluation [19]. By providing a foundation for the objective (quantitative/numerical) assessment of a patient’s condition, these systems can potentially improve communication between health care professionals [2], thus preventing misunderstandings and misinterpretations. Providing nurses and physicians with a tool for reaching a common understanding of the meaning of deviations from normal physiology is potentially advantageous.

This system can be valuable merely as a means of increasing awareness of the importance of repeatedly recording clinical parameters, and of the fact that deviations from normal physiological parameters are actually negative prognostic factors.

As the guidelines for escalation were only introduced in the present study, we do not know the number of patients that received treatment, including escalation to a higher level of care, on the basis of a high PEWS. However, the PEWS did not have an impact on decisions to administer fluid boluses/i.v. fluids or i.v. antibiotics, and we believe that the PEWS at this early stage did not affect decisions to treat patients as in- or outpatients. In fact, we believe that an advantage of our study design is that our patients were included during the first months of introduction of the PEWS, before it was established as an assessment tool and started to influence decisions to deliver interventions like the ones we have examined in this study.

The results of this study do not provide information as to whether the PEWS prevented ICU transfers or further deteriorations. However, the study enabled the establishment of criteria for escalation of care in this context. With regards to inter-rater and intra-rater variability, as well as sensitivity and specificity of the PEWS we plan to perform a prospective multicenter study in order to achieve sufficient power.

## Conclusion

A high PEWS was associated with known negative prognostic factors such as diseases affecting the lower airways and cardiovascular disease, a younger age, and the need for fluid resuscitation and/or oxygen supplementation. Therefore, the modified Brighton PEWS may be a useful tool for the identification of children at high risk of cardio-respiratory deterioration and failure in a department of pediatric and adolescent medicine like ours. A PEWS ≥3 should indicate that careful monitoring of the patient is required.

## References

[1] Subbe CP, Slater A, Menon D, Gemmell L (2006) Validation of physiological scoring systems in the accident and emergency department. Emerg Med J 23: 841–845. 17057134 10.1136/emj.2006.035816 PMC2464409

[2] Gardner-Thorpe J, Love N, Wrightson J, Walsh S, Keeling N (2006) The value of Modified Early Warning Score (MEWS) in surgical in-patients: a prospective observational study. Ann R Coll Surg Engl 88: 571–575. 17059720 10.1308/003588406X130615 PMC1963767

[3] Morgan RJMW, F.; Wright,M.M. (2007) An Early Warning Scoring System for detecting developing critical illness. Clin Intensive Care 8.

[4] Tibballs J, Kinney S (2009) Reduction of hospital mortality and of preventable cardiac arrest and death on introduction of a pediatric medical emergency team. Pediatr Crit Care Med 10: 306–312. 19307806 10.1097/PCC.0b013e318198b02c

[5] Nadkarni VM, Larkin GL, Peberdy MA, Carey SM, Kaye W, et al (2006) First documented rhythm and clinical outcome from in-hospital cardiac arrest among children and adults. JAMA 295: 50–57. 16391216 10.1001/jama.295.1.50

[6] Suominen P, Olkkola KT, Voipio V, Korpela R, Palo R, et al (2000) Utstein style reporting of in-hospital paediatric cardiopulmonary resuscitation. Resuscitation 45: 17–25. 10838235 10.1016/s0300-9572(00)00167-2

[7] Torres A Jr, Pickert CB, Firestone J, Walker WM, Fiser DH (1997) Long-term functional outcome of inpatient pediatric cardiopulmonary resuscitation. Pediatr Emerg Care 13: 369–373. 9434991 10.1097/00006565-199712000-00002

[8] Monaghan A (2005) Detecting and managing deterioration in children. Paediatr Nurs 17: 32–35. 15751446 10.7748/paed2005.02.17.1.32.c964

[9] Akre M, Finkelstein M, Erickson M, Liu M, Vanderbilt L, et al (2010) Sensitivity of the Pediatric Early Warning Score to Identify Patient Deterioration. Pediatrics 125: e763–769. 20308222 10.1542/peds.2009-0338

[10] Sax FL, Charlson ME (1987) Medical patients at high risk for catastrophic deterioration. Crit Care Med 15: 510–515. 3568715 10.1097/00003246-198705000-00012

[11] Smith AF, Wood J (1998) Can some in-hospital cardio-respiratory arrests be prevented? A prospective survey. Resuscitation 37: 133–137. 9715771 10.1016/s0300-9572(98)00056-2

[12] Tobin AE, Santamaria JD (2012) Medical emergency teams are associated with reduced mortality across a major metropolitan health network after two years service: a retrospective study using government administrative data. Crit Care 16: R210. 23107123 10.1186/cc11843 PMC3682314

[13] Slater A, Shann F, McEniery J (2003) The ANZPIC registry diagnostic codes: a system for coding reasons for admitting children to intensive care. Intensive Care Med 29: 271–277. 12541153 10.1007/s00134-002-1600-3

[14] Berens RJ, Cassidy LD, Matchey J, Campbell D, Colpaert KD, et al (2011) Probability of survival based on etiology of cardiopulmonary arrest in pediatric patients. Paediatr Anaesth 21: 834–840. 21199129 10.1111/j.1460-9592.2010.03479.x

[15] Papastamelos C, Panitch HB, England SE, Allen JL (1995) Developmental changes in chest wall compliance in infancy and early childhood. J Appl Physiol 78: 179–184. 7713809 10.1152/jappl.1995.78.1.179

[16] Reis AG, Nadkarni V, Perondi MB, Grisi S, Berg RA (2002) A prospective investigation into the epidemiology of in-hospital pediatric cardiopulmonary resuscitation using the international Utstein reporting style. Pediatrics 109: 200–209. 11826196 10.1542/peds.109.2.200

[17] Van den Bruel A, Aertgeerts B, Bruyninckx R, Aerts M, Buntinx F (2007) Signs and symptoms for diagnosis of serious infections in children: a prospective study in primary care. Br J Gen Pract 57: 538–546. 17727746 PMC2099636

[18] Thompson M, Van den Bruel A, Verbakel J, Lakhanpaul M, Haj-Hassan T, et al (2012) Systematic review and validation of prediction rules for identifying children with serious infections in emergency departments and urgent-access primary care. Health Technol Assess 16: 1–100. 10.3310/hta16150 PMC4781278 22452986

[19] Skaletzky SM, Raszynski A, Totapally BR (2012) Validation of a modified pediatric early warning system score: a retrospective case-control study. Clin Pediatr (Phila) 51: 431–435. 22157421 10.1177/0009922811430342

[20] Ludikhuize J, Smorenburg SM, de Rooij SE, de Jonge E (2012) Identification of deteriorating patients on general wards; measurement of vital parameters and potential effectiveness of the Modified Early Warning Score. J Crit Care 27: 424 e427–413. 10.1016/j.jcrc.2012.01.003 22341727

